# Supplementary material for: Mechanical control of tissue shape and morphogenetic flows during vertebrate body axis elongation
Source: Sci Rep. 2021 Apr 21;11:8591. doi: 10.1038/s41598-021-87672-3 (PMC8060277; doi:10.1038/s41598-021-87672-3)
Supplement: Supplementary file 1 — Supplementary Information 1. [file 41598_2021_87672_MOESM1_ESM.pdf]

Supplementary Information for:

Mechanical control of tissue shape and morphogenetic flows during vertebrate body axis elongation

Samhita P. Banavar, Emmet K. Carn, Payam Rowghanian,
Georgina Stooke-Vaughan, Sangwoo Kim & Otger Campàs
